# The Skin Microbiota of *Eleutherodactylus* Frogs: Effects of Host Ecology, Phylogeny, and Local Environment

**DOI:** 10.3389/fmicb.2019.02571

**Published:** 2019-11-06

**Authors:** Liza Garcia-Recinos, Patricia A. Burrowes, Maria Dominguez-Bello

**Affiliations:** ^1^Department of Biology, University of Puerto Rico, San Juan, Puerto Rico; ^2^Centro de Estudios Conservacionistas, Universidad de San Carlos de Guatemala, Guatemala, Guatemala; ^3^Department of Biochemistry and Microbiology, Rutgers University, New Brunswick, NJ, United States

**Keywords:** amphibian, skin microbiota, ecology, phylogeny, Puerto Rico, 16S rRNA gene

## Abstract

Amphibian skin microbiota has a potential protective role against diseases. However, the effects of environmental and host factors on symbiotic bacterial communities are not well understood. Caribbean frogs in the genus *Eleutherodactylus* represent a case of congeneric species that differ in ecological specialization by the process of adaptive radiation. For a small clade of *Eleutherodactylus* from Puerto Rico, we investigated the role of local environments, host species, and microhabitat in the composition of their skin microbiome. The potential congruence between microbial communities in hosts that are most closely related phylogenetically was also addressed. We hypothesized that the skin microbiota of *Eleutherodactylus* frogs would be mostly associated to microhabitat use, but also differ according to locality, and to a lesser extent to host species. To test this hypothesis, we swabbed the skin of a total of 98 adult individuals of seven *Eleutherodactylus* species distributed in two nearby localities in Puerto Rico, and sequenced the V4 region of the 16S rRNA gene. Results showed that locality had the greatest effect on determining skin bacterial communities of amphibian hosts, but this effect was stronger on the composition (based on presence/absence) than on its structure (based on sequence abundance). The most ecologically distinct host, *E. cooki*, and the generalist *E. coqui* presented, respectively, the most dissimilar and similar microbiota compared to other hosts. Host phylogeny showed a weak influence on skin microbiota. Results suggest that both local environment and ecological specialization are structuring the skin bacterial community in these *Eleutherodactylus* species, but that characteristics intrinsic to species may also render unique hosts the ability to maintain distinct microbiotas.

## Introduction

Diverse microbial communities inhabit animal and plant hosts and may play a major role in host processes from nutrition (Turnbaugh et al., 2006) and tissue development (Koropatnick et al., 2004), to immune system modulation (Mazmanian et al., 2005). The study of microbial communities residing on amphibian skin has received a lot of attention lately as some bacteria produce antifungal metabolites that may render protection against the pathogenic chytrid fungus *Batrachochytrium dendrobatidis* (*Bd*) (Woodhams et al., 2007; Harris et al., 2009; Becker et al., 2015a, b; Catenazzi et al., 2017). This fungus produces the skin infection chytridiomycosis and has been associated to extinctions and population declines in many amphibian species all over the world (Berger et al., 1998; Rachowicz et al., 2006; Skerratt et al., 2007; Scheele et al., 2019). Studies have shown that susceptibility to *Bd* is associated to variation in skin microbiota structure in some amphibian species and populations (Lam et al., 2010; Becker et al., 2015b; Longo and Zamudio, 2017a). Microbial communities in amphibian skin can vary in association to several factors such as host species (McKenzie et al., 2011; Kueneman et al., 2013; Belden et al., 2015), populations within species (Rebollar et al., 2016; Hernández-Gómez et al., 2017; Hughey et al., 2017; Longo and Zamudio, 2017b; Prado-Irwin et al., 2017; Muletz Wolz et al., 2018), pathogens (Jani and Briggs, 2014; Longo and Zamudio, 2017a), developmental stage (Kueneman et al., 2013; Longo et al., 2015; Sabino-Pinto et al., 2017) and season (Longo et al., 2015), among others. However, the influence of host evolutionary processes on skin microbiota had not been considered until more recent studies that include host phylogeny (Bletz et al., 2017a; Bird et al., 2018). Caribbean frogs in the genus *Eleutherodactylus* are an example of adaptive radiation where more than 160 species exist and often occupy different microhabitats within an island (Hedges, 1989; Hedges et al., 2008). Examples of replicate radiations have shown that species confronting similar selective pressures at particular niches have evolved paralleled phenotypes (Losos et al., 1998). A recent study found that *Eleutherodactylus* living in different microhabitats have significantly different morphologies and that these were convergent across the Caribbean, underscoring the effect of similar ecological niches on evolutionary outcomes for this group (Dugo-Cota et al., 2019). The aim of this study is to tease apart the relative contribution of (1) local environments (locality effect), (2) host species (host species effect), and (3) microhabitat (ecological effect) in the composition of the skin microbiome of a small clade of Puerto Rican *Eleutherodactylus*. In addition, we test the hypothesis that hosts that are more closely related phylogenetically, would have most similar microbial communities (potential phylogeny effect). Despite research advances, the relative importance of these variables in driving variability of skin microbial communities is not well understood.

Ecologically similar hosts can differ in skin microbiota composition and structure when distantly related (McKenzie et al., 2011; Bletz et al., 2017d), however host relatedness and ecology are usually confounded factors (Kueneman et al., 2013; Walke et al., 2014; Bletz et al., 2017d; Muletz Wolz et al., 2018). Host specificity of the amphibian microbiota might be attributable in part to specific chemical composition of skin secretions that might select for particular microorganisms (Woodhams et al., 2014), as antimicrobial peptides do in the cnidarian genus *Hydra* (Franzenburg et al., 2013). Skin morphology and chemistry could be associated to phylogeny, as some lineages of amphibians might secrete similar components, and to ecology, as microhabitat conditions could select for similar skin or physiological characteristics in the host. In fact, more recent studies show amphibian ecology as an important factor shaping skin microbiota (Belden et al., 2015; Bletz et al., 2017a; Sabino-Pinto et al., 2017), while host phylogeny does not seem to influence it as strongly (Bletz et al., 2017a; Bird et al., 2018). Efforts to discern the effects of host locality, microhabitat use, or relatedness on similarity of microbial communities are important biologically because they may reveal alternate evolutionary or ecological pathways that confer hosts advantages leading to local-adapted ecomorphs, or resistance to novel pathogens. For example, a strong microhabitat effect on the skin microbiome among ecomorphs of *Eleutherodactylus* in the Caribbean, would suggest that host skin physiology and its capacity to maintain certain symbionts, is another host trait associated with ecological diversification.

Members of the genus *Eleutherodactylus* are direct-developing frogs (lack an aquatic larval phase) and most species provide parental care by tending terrestrial eggs (Joglar, 1998). In mammals and termites, both host specificity and phylogenetic congruence with host microbiome are attributed in part to parental care and other behaviors that enable vertical inheritance of gut symbionts through evolutionary time (Ley et al., 2008; Ochman et al., 2010; Abdul Rahman et al., 2015). In amphibians with parental care, transmission of bacteria from parents to eggs has been suggested (Banning et al., 2008; Walke et al., 2011). The amphibian community in Puerto Rico is mostly composed by *Eleutherodactylus* frogs, so that the majority, or all the species co-occurring at any given locality are congeneric and endemic (Rivero, 1978). These species share reproductive mode, developmental traits, ontogeny, and a relatively recent evolutionary history (Joglar, 1989; Pyron and Wiens, 2013) minimizing variation due to these factors.

Herein, we characterized the skin microbiota of seven *Eleutherodactylus* species distributed in two nearby localities. Because there are differences in the microhabitats where these species are active at night (Joglar, 1998) we hypothesized that influence of host microhabitat (ecology) on skin microbiota composition and structure would be stronger than that of locality or host species. Based on results from previous work with *E. coqui* in an altitudinal gradient (Hughey et al., 2017), we expected that locality would come second at explaining further variation. Lastly, because our sample included closely related congeners, we presumed that host phylogeny would have the least influence on microbial communities but predicted greater similarity among closely related hosts compared to those more phylogenetically distant. Therefore, our study aimed to unravel how host endogenous and exogenous factors influence the variation in the composition (bacteria presence/absence) and structure (considering also bacterial abundance) of skin microbiota.

## Materials and Methods

### Field Sampling

We sampled seven *Eleutherodactylus* species (Table 1) that occurred in two nearby localities in the Cordillera de Cayey in central-east Puerto Rico. One widely distributed and ecological generalist species (*E. coqui*), was sampled at both localities to control for site effects. We will refer to these two localities as Charco Azul (18°05′25.51″N, 66°01′57.56″W), which is located at 605 masl, in the protected area of Carite National Forest, and “Quebrada” (18°02′59.57″N, 65°59′23.95″W), at 360 masl, located just outside the protected area alongside the road 181. Both localities are classified in the subtropical wet forest ecological life zone by the Holdridge system (Ewel and Whitmore, 1973) and are separated by a linear distance of only 6.4 kilometers. There is a small stream at both localities, but the one in Quebrada contains large boulders of granite rocks with cracks and crevices inhabited by a very specialized species, *E. cooki* (Burrowes, 2000). Although both localities are accessible to visitors, the Quebrada site is unprotected and shows more signs of disturbance. For each host species and both populations of *E. coqui*, 15 adult individuals were sampled (*N* = 121) in a period of 3 consecutive days at the end of March 2017 to minimize temporal variation in skin communities (Sabino-Pinto et al., 2017).

**TABLE 1:** Number of samples per host species studied at each locality and corresponding nocturnal microhabitat.

**Host species**	**Sample size after rarefaction**	**Locality**	**Microhabitat**
*E. brittoni*	9	Charco Azul	herbaceous/open areas
*E. coqui*	10	Charco Azul	Arboreal
*E. locustus*	15	Charco Azul	Arboreal
*E. wightmanae*	13	Charco Azul	Terrestrial
*E. portoricensis*	13	Charco Azul	Arboreal
*E. coqui*	11	Quebrada	Arboreal
*E. richmondi*	13	Quebrada	Terrestrial
*E. cooki*	14	Quebrada	Cave/rocks
Environment	20	Charco Azul	Leaf litter, leaves, trunk, bromelia
Environment	20	Quebrada	Leaf litter, leaves, trunk, rock

Surveys were conducted at night when *Eleutherodactylus* are active. Frogs were captured using a clean pair of nitrile gloves to avoid microbial cross contamination among individuals, and kept in individual sterile Whirl-Pak bags until sampling. Prior to taking samples, frogs were rinsed with 50 ml sterilized water to clear the skin from soil and the majority of transient bacteria (those not necessarily associated to host’s skin) (Lauer et al., 2007). Immediately after this, a sterile rayon swab (MW113; Medical Wire & Equipment, Corsham. Wiltshire, United Kingdom) was passed over the ventral surface of the frog 10 times, and 5 times in each hind and front leg (including the foot), for a total of 30 times per individual. Each swab was kept in 1.5 ml sterile vials in liquid nitrogen until storage in a −80°C freezer. Environmental samples (*N* = 40, Table 1) were taken from nocturnal perching sites and most probable diurnal refuges to compare samples from amphibian microhabitats to host skin.

### DNA Extraction and Sequencing Methods

DNA was extracted from swabs using the MoBio (CA, United States) PowerSoil-htp 96-well soil DNA isolation plates, according to the instructions provided by the manufacturer. Extracted DNA from samples was stored at −80°C until sequencing. The V4 region of 16s rRNA gene was amplified by PCR using barcoded primers, as described by Caporaso et al. (2012). Studies on amphibian skin microbiome usually do not include control samples or do not report the results of these samples. However, bacterial communities of amphibian skin include environmentally related OTUs, and several of these have been reported as core or abundant OTUs in amphibians (McKenzie et al., 2011; Loudon et al., 2014; Rebollar et al., 2016), which also correspond to reported contaminating genera on DNA extraction kits (eg. *Pseudomonas, Sphingomonas, Stenotrophomonas, Janthinobacterium*, most belonging to the phylum Proteobacteria (Salter et al., 2014; Jervis-Bardy et al., 2015). To control for possible contamination, blank swabs from the laboratory and field air, and the water used for rinsing the frogs were also included in the extraction as controls (Supplementary Table S1). These control samples as well as reagents for DNA extraction and PCR amplification were also sequenced (Salter et al., 2014). The amplicons were pooled in equimolar ratios and purified with QIAquick PCR purification kit (Qiagen Inc., CA, United States). Sequencing of pooled amplicons were done on Illumina MiSeq platform (Genome Technology Center of NYU Medical Center, NY) using a paired-end technique (2 150-cycle runs).

### Sequences Processing

Sequences were processed using QIIME pipeline v1.9.0 (Caporaso et al., 2010), assembling forward and reverse reads and demultiplexing samples. Sequences were quality filtered (including chimera filtering) using default settings. The open reference protocol was used to cluster sequences into Operational Taxonomic Units (OTUs) at 97% similarity threshold with the USEARCH method (Edgar, 2010) and the 13.8 version of the Greengenes reference database (McDonald et al., 2011), sequences that did not match the database were clustered as *de novo* OTUs. Taxonomy was assigned based on the Greengenes database using the RDP Classifier (Wang et al., 2007). Representative sequences were aligned to the Greengenes reference using PyNAST (Caporaso et al., 2010) to build a phylogenetic tree with RAxML (Stamatakis, 2006). All chloroplast and mitochondrial OTUs were removed, as well as OTUs representing less than 0.001% of total counts (Bokulich et al., 2013). OTUs present in control samples (field and lab blank swabs, rinsing water, DNA extraction and PCR; Supplementary Table S1) were considered contaminants if more prevalent and equally or significantly more abundant on control samples than on skin samples (Lazarevic et al., 2014; Salter et al., 2014; Troccaz et al., 2015), based on a Kruskal-Wallis test. These OTUs were removed from the OTU table (Supplementary Table S2). We consider this approach more appropriate than removing all OTUs present in control samples as cross-contamination from study samples to control samples can occur (Salter et al., 2014). Rarefaction of OTU table for the downstream analyses was performed at 3,000 sequences per sample.

### Data Analysis

Analysis were performed with three objectives: (1) to compare the bacterial communities in the environment versus those found in frog skin, (2) to assess the influence of locality, microhabitat use and host species on amphibian skin microbiota, and 3) to evaluate the congruence of host phylogeny with similarity of bacterial communities on frog skin.

To compare the bacterial communities in the environment with those in frog skin, we estimated Phylogenetic Diversity (PD) as a measure of alpha diversity and assessed differences with a non-parametric *t*-test. Beta diversity was calculated with the phylogenetic based distances Unifrac, both unweighted (presence/absence data) to assess community composition, and weighted (which also takes into account abundance data) to assess structure. Differences in beta diversity between categories were assessed with Analysis of Similarity (ANOSIM) on 999 permutations and principal coordinate analysis was used to visualize patterns. Then, we used the Linear Discriminant Analysis Effect Size (LEfSe) software (Segata et al., 2011) to determine differential OTUs between categories considering only OTUs with LDA score >3.0.

To assess the effect of locality, microhabitat use and host species on skin associated bacteria, we estimated alpha diversity of bacterial communities using Phylogenetic Diversity (PD) and Shannon diversity index. We used general linear models with these alpha diversity metrics separately as response variables, to assess the effects of locality and microhabitat, including host species as a random effect, as this factor was nested in locality and microhabitat. Beta diversity was calculated with unweighted and weighted Unifrac distances. We compared the effect of locality, microhabitat use and host species by looking at the principal effect of each variable separately using ANOSIM and PERMANOVA tests. These effects were also explored in a PERMANOVA model, using the Adonis function in the vegan package (Oksanen et al., 2017). Principal effects were evaluated by sequentially adding factors, with the model formula “beta diversity metric∼Locality + Microhabitat + Species” since host species was nested in the other two factors. While ANOSIM and PERMANOVA tests ran for each factor separately were somewhat in agreement, the PERMANOVA model produced different results that may be associated to the way in which variables are ordered (Supplementary Table S5). Following significant differences in alpha or beta diversity among categories, *post hoc* pairwise comparisons were performed, with False Discovery Rate correction for multiple comparisons (Benjamini and Hochberg, 1995). We used the Linear Discriminant Analysis Effect Size (LEfSe) software (Segata et al., 2011) to determine differential OTUs between categories considering only OTUs with LDA score >3.0. A core microbiota was determined for each amphibian host species or population, considering the OTUs present in ≥90% of individuals for a given category.

To assess congruence of skin microbial communities to host phylogeny, we constructed dendrograms based both on unweighted and weighted Unifrac distances. OTU tables were collapsed by host species (Brooks et al., 2016; Bletz et al., 2017a), or populations for *E. coqui* since it was present at both localities, and dendrograms were constructed by unweighted pair group method with arithmetic mean (UPGMA) using jackknife subsampling (jackknife_beta_diversity.py) with 100 replications. The phylogenetic tree for the *Eleutherodactylus* frogs studied was extracted from Pyron and Wiens (2013), with non-target species pruned from the tree. Topological congruence between microbiota dendrograms and host phylogeny was assessed using the normalized Robinson-Foulds distance, which value ranges from 0 (complete congruence) to 1 (no congruence). Significance for this distance was obtained creating 1,000 random trees with the same number of tips as the dendrograms, and topological congruence of each random tree with host phylogeny was estimated. The probability to obtain a tree with the same or better congruence than the corresponding microbiota dendrogram determined the significance (Brooks et al., 2016).

Since locality effect was apparent in the dendrograms constructed, we also evaluated whether host species clustering by UPGMA was influenced by locality and/or by microhabitat use. We applied phylogenetic eigenvector regressions (PVR) via tree decomposition (Diniz-Filho et al., 1998) to both dendrograms based on Unifrac distances. While the PVR method was originally proposed for phylogenetic trees, we adapted it here for using dendrograms given their tree-like structure. To assess whether locality and microhabitat significantly predicted structure in the dendrograms, their effect was explored using a multivariate analyses of variance (MANOVA). The PVR analysis was conducted in R (R Core Team, 2014) using the packages ape (Paradis et al., 2004) and phytools (Revell, 2012). To further evaluate the influence of host phylogeny in the skin microbiota using all species studied, we tested for correlations between phylogenetic and Unifrac distances using a partial Mantel test, controlling for locality. Matrices with averaged unweighted and weighted Unifrac distances by host species and population were used. Phylogenetic distances among hosts were calculated as patristic distances from the host phylogenetic tree using the function cophenetic.phylo from the ape package (Paradis et al., 2004).

## Results

We analyzed skin samples collected from 121 adult frogs and from 40 environmental samples (Table 1). After filtering OTUs for quality processing and removing OTUs from control samples (Supplementary Tables S1, S2 and Supplementary Figure S1), a total of 1,158,330 sequences were obtained from all samples, yielding 5,453 OTUs. After rarefying at 3,000 sequences per sample (with 23 frog samples from different species lost due to low coverage), a total of 5,323 OTUs remained (average-frogs: 283 ± 68 OTUs per sample, environment: 529 ± 119 per sample; Supplementary Table S3).

### Bacterial Communities Differed Between Frog Skin and the Environment

**FIGURE 1:**
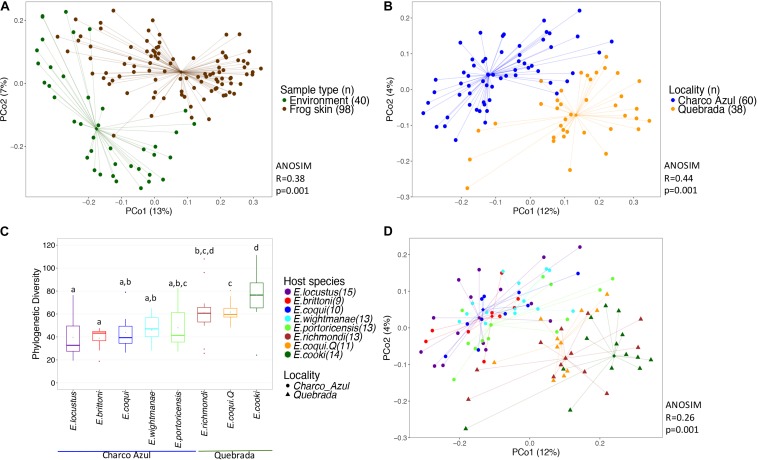
Diversity of bacterial communities in the skin of *Eleutherodactylus* frogs and the environment. **(A)** PCoA of Unifrac distances between frog skin and environmental bacteria, **(B)** PCoA of frog skin microbiota by locality, **(C)** Phylogenetic alpha diversity of bacterial communities in frogs’ skin per host and locality. Gray points within each boxplot correspond to the mean value. Different letters indicate a significant difference (*p* < 0.05). **(D)** PCoA of skin microbiota by frog species. All PCoA’s are based on unweighted Unifrac distances. Lines connect each sample to its group spatial median (diamond shaped).

Bacterial communities on frog skin differed significantly from those in the environment, based on Unifrac distances, both unweighted (ANOSIM: *R* = 0.36, *p* = 0.001, Figure 1A) and weighted (ANOSIM: *R* = 0.38, *p* = 0.001, Supplementary Figure S2a). Bacterial phylogenetic diversity in frog skin was lower than in environmental samples (*t*-test: *t* = 6.77, *p* = 0.001, Supplementary Figure S3a). Frog skin was enriched in Gammaproteobacteria, Betaproteobacteria, Clostridia, Bacilli and Sphingobacteriia, while environmental samples were enriched by a greater number of bacterial taxa including Alphaproteobacteria, Spartobacteria, Saprospirae, Actinobacteria and Acidobacteria among others (Supplementary Figure S3b and Supplementary Table S4).

### Factors Influenced in Different Ways the Diversity, Composition, and Structure of Skin Bacterial Communities

#### Microbial Diversity

Alpha diversity of skin bacterial communities measured as phylogenetic diversity varied among localities and the interaction microhabitat:caves/rock (GLM, *p* < 0.001 and *p* < 0.05, respectively, Figure 1C), while Shannon diversity varied only between localities (GLM, *p* < 0.001, Supplementary Figure S2c). Pairwise comparisons among hosts showed that *E. cooki* was distinguished by having significantly higher bacterial phylogenetic diversity than all other hosts (pairwise KW, all *p* < 0.05, Figure 1C), with exception of *E. richmondi* (*p* < 0.05). Comparing between *E. coqui* populations, individuals from Quebrada, had higher bacterial phylogenetic diversity than individuals from Charco Azul (KW, *p* = 0.012). For Shannon diversity however, the locality effect was driven only by the significant difference between *E. cooki* and *E. locustus* (KW, *p* < 0.05, Supplementary Figure S2c).

#### Microbial Composition

With respect to the composition of the microbiota (presence or absence of OTU’s), locality had the most important effect (ANOSIM *R* = 0.44, *p* < 0.001, Figure 1B), followed by host species (ANOSIM *R* = 0.26, *p* < 0.001, Figure 1D) with a milder effect. Microhabitat was only significant based on individual PERMANOVA (Supplementary Table S5). *Post hoc* pairwise comparisons between host species underscore that the higher differences in skin microbiota composition, were driven by hosts between localities (unweigthed Unifrac, pairwise ANOSIM: all *p* < 0.005, Table 2a, and Figure 1D). Within Charco Azul, only *E. locustus-E. portoricensis* microbiota differed significantly (ANOSIM: *p* = 0.036).

**TABLE 2:** Pairwise comparisons of (a) unweighted and (b) weighted Unifrac distances among skin bacterial communities of *Eleutherodactylus* species at two localities showing *R* values from ANOSIM.

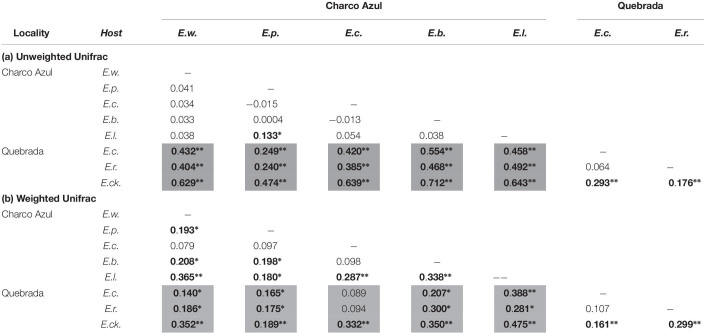

*Values in bold indicate significant differences (FDR corrected *p*-values): ^∗^*p* < 0.05, ^∗∗^*p* < 0.01. Shaded area corresponds to comparisons of hosts between localities.*

The observed secondary effects of amphibian host-species and that of microhabitat on bacterial composition may be attributed to the presence of unique species like *E*. *cooki* in the community of hosts. This species is an ecological specialist that lives exclusively in caves or within rock crevices along stream banks. *Post hoc* pairwise comparisons also showed that in fact the microbiota composition of *E. cooki* was highly distinct from all other hosts regardless of locality (ANOSIM (R) Table 2a, or PERMANOVA (Pseudo-F) Supplementary Table S6). The only exception was obtained from PERMANOVA *R*^2^ values which showed non-significant differences between the microbiota of *E. cooki* and the two other species from the same locality of Quebrada (Supplementary Table S6).

#### Microbial Structure

Interpretation of the analyses of the effect of locality, microhabitat and species in the structure of the skin microbiota among the amphibians studied, proved to be more challenging because the results varied depending on the test or model applied. Individual ANOSIM and PERMANOVA tests to evaluate principal effects showed that the effect of host species is greatest (*R* = 0.28, *p* < 0.001; *R*^2^ = 0.21, *p* < 0.001), followed weakly by microhabitat (*R* = 0.081, *p* < 0.04; *R*^2^ = 0.10, *p* < 0.001); and then by locality (*R* = 0.03, *p* < 0.056; *R*^2^ = 0.0439, *p* < 0.001) (Supplementary Table S5). However, in the PERMANOVA model with all factors, only locality was significant when considering microbiota structure. This is probably caused by the fact that factor ordering matters in these models, and also because in this study the locality effect is inherent in the species effect, as different amphibian communities were present in each locality.

*Post hoc* pairwise comparisons between host species via weighted Unifrac distances using ANOSIM, showed that most species differed in structure of microbiota even within the same locality (Table 2b), although this was visually less evident in the principal coordinate analysis (Supplementary Figures S2b,d), than the locality effect (Figure 1B). In this analysis the ecological generalist and widely distributed *E. coqui*, was the most similar to other hosts within the same locality, differing significantly only from *E. locustus* at Charco Azul, and from *E. cooki* at Quebrada (pairwise ANOSIM, both *p* < 0.01). Moreover, *E. locustus* and *E. cooki* differed the most in skin microbiota structure compared to other hosts, regardless of locality (Table 2b). As with unweighted Unifrac distances, ANOSIM and PERMANOVA (Pseudo-F) statistics gave similar results, while those obtained from the PERMANOVA (*R*^2^) were very different (Supplementary Table S7).

**FIGURE 2:**
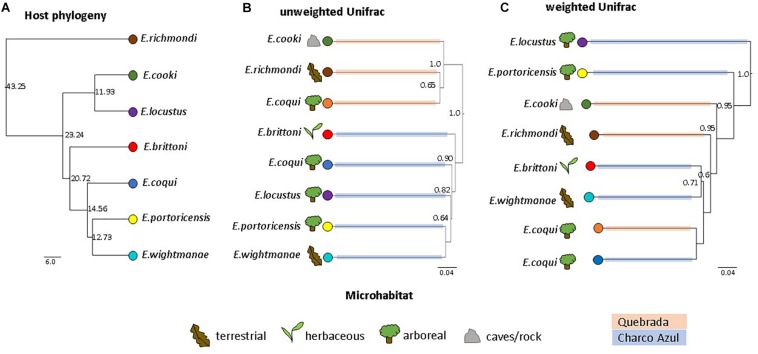
Comparison of host phylogeny and skin microbiota dendrograms. **(A)** Host phylogeny, node values indicate millions of years. **(B)** UPGMA clustering of unweighted, and **(C)** weighted Unifrac distances among skin bacterial communities of *Eleutherodactylus* species. Node values correspond to jackknife support values (values ≥0.5 are shown). Figures represent host microhabitat and colored branches in microbiota dendrograms correspond to host locality. UPGMA clustering shows a strong effect of locality on unweighted Unifrac distances, while clustering based on weighted distances is not influenced by locality nor microhabitat. The influence of host phylogeny on skin microbiota similarity is not evident.

The UPGMA dendrogram, based on unweighted Unifrac distances collapsed by host species/population, also revealed that microhabitat was not a significant factor structuring the composition of frog skin microbiota, while confirming that it was mainly differentiated by locality (MANOVA: locality, *p* < 0.001; microhabitat, *p* = 0.351; Figure 2B). On the other hand, the dendrogram based on weighted Unifrac distances showed neither clustering by locality nor microhabitat (MANOVA: locality, *p* = 0.862; microhabitat, *p* = 0.860; Figure 2C).

#### Discriminant and Core Skin Bacterial Taxa Among *Eleutherodactylus*

**FIGURE 3:**
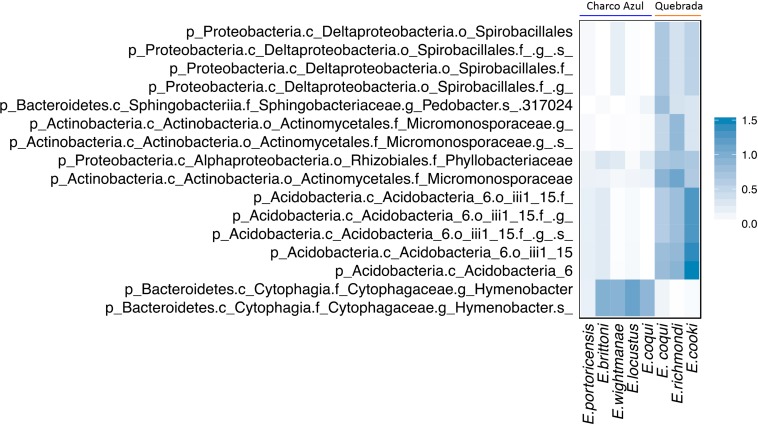
Discriminant skin bacterial taxa between localities for all *Eleutherodactylus* host species, based on Linear Discriminant Analysis (LDA score >3.0). Average abundances by host species are presented. The scale indicates the range of relative abundance in percentages.

We found 16 discriminant skin bacterial taxa by locality. *Eleutherodactylus* skin microbiota at Charco Azul was enriched by two taxa from the genus *Hymenobacter* (Bacteroidetes), while at Quebrada, 14 bacterial taxa were more abundant, mostly from phyla Proteobacteria (Deltaproteobacteria), Bacteroidetes (Sphingobacteriia), Actinobacteria and Acidobacteria (Figure 3). Locality discriminant taxa had a relative abundance of <1% in frog skin (mean percentage ± sd: 0.8 ± 0.4 at Charco, 0.7 ± 0.3 at Quebrada), and together represented less than 1.5% of the microbiota for a host species.

**FIGURE 4:**
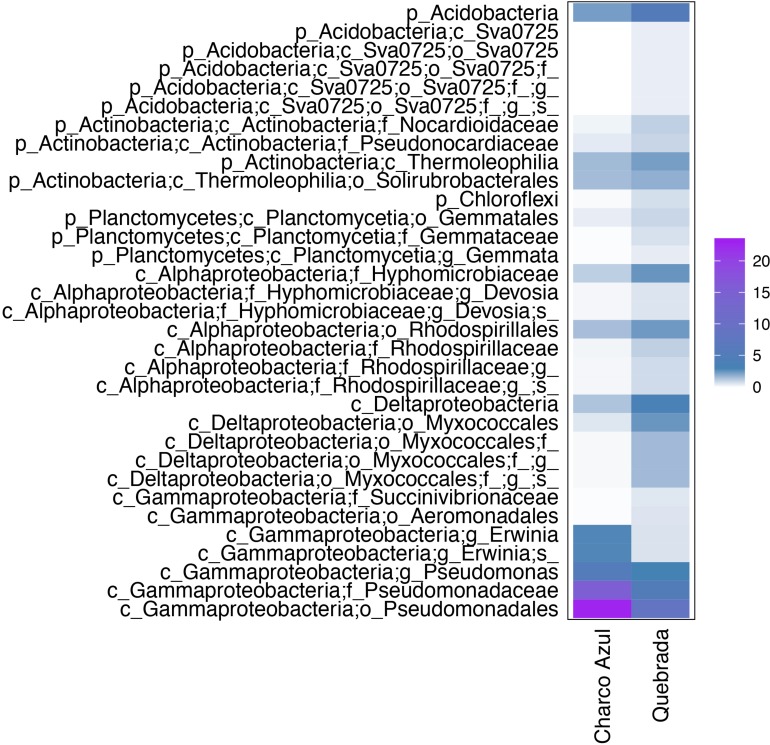
Differentially abundant bacterial taxa in the skin of two populations of *E. coqui*. Based on Linear Discriminant Analysis (LDA score >3.0). Average abundances by host population are presented. The scale indicates the range of relative abundance in percentages.

At the intraspecific level, when comparing the two populations of *E. coqui*, we found 45 differential OTUs between sites. While skin microbiota of *E. coqui* at Charco Azul was enriched by 10 OTUs in phyla Proteobacteria (Gammaproteobacteria) and Bacteroidetes (Cytophagia), conspecifics at Quebrada were enriched in 35 OTUs mostly in phyla Proteobacteria (Alphaproteobacteria and Deltaproteobacteria), Bacteroidetes (Saprospirae), Actinobacteria and Acidobacteria (Figure 4). Locality discriminant OTUs for *E. coqui* had a relatively high abundance in their skin (mean percentage ± sd: 9.9 ± 8.8 at Charco, 1.3 ± 1.2 at Quebrada) representing up to 22.7% of the microbiota for a given population. The bacterial taxa that presented the highest effect sizes (LDA > 4.0) corresponded to pseudomonads and were more abundant in the skin of frogs at Charco Azul.

**FIGURE 5:**
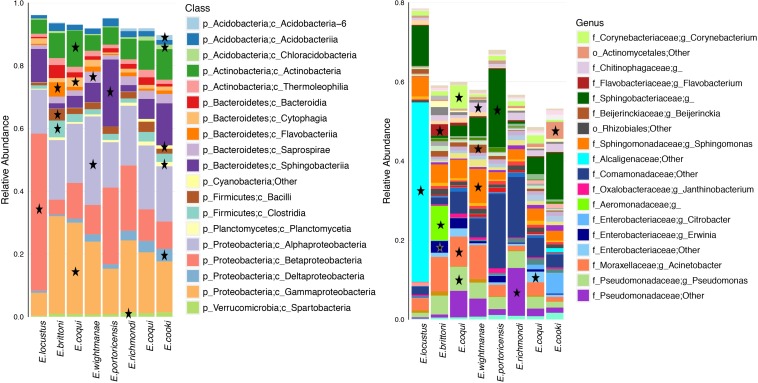
Average relative abundance of bacterial taxa for each host species or population. Bacterial class and genus levels are shown. A star indicates overrepresented bacterial taxa in that specific frog species in relation to all other hosts (LDA score ≥3.0). Bacterial taxa with an average abundance >1% for a given host species are included.

Differentially abundant bacterial taxa for each host species summed a total of 219 (based on a LDA score ≥3.0) (Figure 5 and Supplementary Table S8). Many of these bacterial taxa (47%) belonged to the phylum Proteobacteria. *E. cooki* had the most diverse and highest number of differential OTUs (mainly from phyla Acidobacteria and Proteobacteria) while *E. locustus* had the lowest number (mainly Betaproteobacteria). The number of differential OTUs per host species was *E. cooki* = 50, *E. wightmanae* = 36, *E. coqui*-Charco = 26, *E. coqui*-Quebrada = 28, *E. richmondi* = 25, *E. brittoni* = 23, *E. portoricencis* = 18 and *E. locustus* = 11 (for details see Supplementary Table S8). The core OTUs for each host species summed a total of 62 (range of 13–22 OTUs by host species), 89% being Proteobacteria, of which 55% corresponded to Gammaproteobacteria (Supplementary Table S9). *E. coqui* presented 16 core OTUs found in both populations, and all were Proteobacteria. Only 7 OTUs comprised a core microbiota across all host species studied: 1 Pseudomonadaceae, 1 *Erwinia*, 4 *Pseudomonas* and *P. veronii* (Supplementary Table S9).

#### Phylogeny Had a Weak Influence on Skin Bacterial Communities

Because of locality effects, topological congruence between host phylogeny and microbiota dendrograms was estimated separately for each locality. For the species in Charco Azul, both Unifrac dendrograms had a small but significant congruence to host phylogeny (both normalized Robinson-Foulds distance = 0.67, *p* = 0.04) (Supplementary Figures S4a–c). Clustering of host species within Quebrada was less informative with only 3 host species present and topological congruence was non-significant (Supplementary Figures S4d–f). When considering all host species and accounting for locality, host phylogenetic distances and microbiota similarity were not correlated (partial Mantel test, unweigthed: *r* = 0.25, *p* = 0.22; weighted *r* = −0.11, *p* = 0.57). However, after removing the most ecologically specialized host, *E. cooki*, that was also distinguished by having the most dissimilar skin microbiota, a moderate correlation was significant for unweighted (*r* = 0.62, *p* = 0.02) but not for weighted (*r* = −0.11, *p* = 0.54) Unifrac distances. The lack of phylogenetic congruence in the composition of the skin microbiota is especially evident when samples from the same species, *E. coqui* from different localities, fail to cluster together based on unweighted Unifrac distances (Figure 2). Although the dendrogram based on weighted Unifrac distances does cluster both populations of *E. coqui*, their level of divergence is substantial.

## Discussion

### Locality Effect

Our work reveals that variation in the composition (presence/absence) of frog skin microbiota was strongly dependent on locality, even though the sites considered in this study are very close in linear distance (6.4 km apart). This influence of locality has been found among other populations of *Eleutherodactylus coqui* (Hughey et al., 2017; Longo and Zamudio, 2017b) and other amphibian species (Kueneman et al., 2013; Walke et al., 2014; Rebollar et al., 2016; Hernández-Gómez et al., 2017; Prado-Irwin et al., 2017; Muletz Wolz et al., 2018), at much further distances. Local variation in habitat structure (e.g., bromeliads are present in Charco Azul but not in Quebrada, and high abundance of bamboo only at Quebrada) might reflect subtle environmental differences, that can affect local microbiota or the way amphibians interact with it. Climatic factors can affect amphibian immunity (Raffel et al., 2006), and environmental bacteria (Singh et al., 2014). In addition, seasonality was also found to be a significant driver of *E. coqui* skin microbiota structure (Longo et al., 2015), supporting an effect of climatic conditions on host physiology and/or on environmental sources of bacteria. Despite the influence of locality we found that bacterial communities on frog skin differed from those in the environment and had lower diversity, as found previously (Walke et al., 2014; Rebollar et al., 2016; Bletz et al., 2017a; Prado-Irwin et al., 2017). Interestingly, frog skin also harbors less diverse bacterial communities than sympatric reptiles in an Australian community (Weitzman et al., 2018). Thus, our results support previous findings showing that environmental communities are an important source of bacteria for amphibians (Loudon et al., 2014), but also that skin mucous and secretion of antimicrobial peptides probably serve as a filter of environmental bacteria (Walke et al., 2014).

### Host-Species Effect

For a given site, host species showed little differences on the composition of skin bacteria (Table 2a), however, most host species did differ subtly but significantly in skin microbiota structure (Table 2b). Recent studies have found that congeneric host species with probably similar ecological habits, did not differ in structure of skin bacteria when sympatric (Bletz et al., 2017d; Muletz Wolz et al., 2018). On the other hand, co-occurring species with very distinct ecology (aquatic, terrestrial or arboreal), or ecologically similar but distantly related (between amphibian orders or families), can differ in skin bacterial composition and/or structure (McKenzie et al., 2011; Kueneman et al., 2013; Walke et al., 2014; Belden et al., 2015; Bletz et al., 2017d). The species considered in this study are all closely related congeners that differ somewhat in habitat use (Table 1). However, contrary to our original hypothesis, we found that skin bacterial communities in *Eleutherodactylus* species did not cluster by general microhabitat use (e.g., arboreal species like *E. coqui* and *E. portoricensis*, versus terrestrial species like *E. wightmanae* and *E. richmondi*). A similar result was found for sympatric reptiles and congeneric frogs in Australia, where skin microbiota did not differ accordingly to host microhabitat (Christian et al., 2018; Weitzman et al., 2018). One explanation could be that these hosts differ in physiology or immunity, which in turn, could enhance growth of particular bacterial taxa (Woodhams et al., 2014). Alternatively, microhabitat use in these *Eleutherodactylus* frogs may differ more than their general classification as arboreal, low understory or terrestrial, since diurnal refuges can be different from nocturnal ones, as well as the sites for egg deposition (Joglar, 1998).

In a diverse community of amphibians in Madagascar, spanning a larger taxonomic and spatial scale, amphibian ecology had a stronger effect on skin bacteria abundance than on its presence (Bletz et al., 2017a). Thus, host factors influencing colonization of skin by bacteria might be more homogenous among amphibians, than factors influencing abundance of bacteria. This suggests that skin microbiota structure might be more species-specific than its composition in these frogs. Further supporting this, is the fact that the two populations of *E. coqui* were more similar based on the structure of their skin bacteria, than on the particular OTU’s present (Table 2). A similar pattern of lower intraspecific variation in skin microbiota structure than in composition has been found for other amphibian hosts, even among populations located much farther away than the ones studied here (Hernández-Gómez et al., 2017; Prado-Irwin et al., 2017; Muletz Wolz et al., 2018). This highlights the fact that the components of microbial communities (presence/absence and abundance) may be influenced by alternative factors or differentially affected by the same factors (Lozupone et al., 2007). Hence it is important to consider both structure and composition of microbial communities in order to better understand the processes and scales at which different factors operate in the assemblage and maintenance of host associated microbial communities. For example, if we are interested in promoting anti-pathogenic properties of bacterial metabolites (e.g., anti-*Bd*), we may want to target the abundant bacteria symbionts, and thus, consider factors that affect the structure versus composition of the microbiome. This study is limited by the correlative nature of the study. Experiments conducting reciprocal transplants of specific bacterial community would be a valuable opportunity to test host specificity of the microbiota.

### Microhabitat Effect

Although microhabitat use was not a strong factor, ecological specialization of the host may influence the bacterial communities in these frogs. For example, *E. cooki* had the most dissimilar skin community both in composition and structure with respect to all other species. At this site, *E. cooki* occurs within rock crevices alongside streams while all other hosts are in contact with vegetation and soil surfaces. Skin microbiota of *E. locustus* also differed strikingly from other hosts due to an overabundance of OTUs in the Family Alcaligenaceae; but we cannot explain this distinctiveness of *E. locustus* from an ecological perspective, because other hosts (like *E. brittoni* and to a certain extent *E. coqui*) also utilize low understory vegetation in the forest. Thus, this suggests that in some cases, host skin microbiota can reflect some physiological restriction or ecological distinctiveness beyond the scope of this study. On the other hand, skin microbiota structure of the most generalist and widely distributed host, *E. coqui*, was the most similar with the rest of hosts. This is expected because *E. coqui* occupies a variety of microhabitats (Joglar, 1998) that could differ in physical structure and microclimatic conditions and thus, affect available microbial pools.

### Discriminant and Core Skin Bacterial Taxa Among *Eleutherodactylus*

For the *Eleutherodactylus* studied here, most of the differentially abundant and core skin bacteria were representatives of Proteobacteria, specially pseudomonads (class Gammaproteobacteria). These are common and usually abundant on amphibian skin, including *E. coqui* (Belden et al., 2015; Walke et al., 2015; Hughey et al., 2017; Longo and Zamudio, 2017b; Prado-Irwin et al., 2017), and comprise a high proportion of members with antifungal properties (Becker et al., 2015b; Woodhams et al., 2015; Bletz et al., 2017b, c). A higher abundance of pseudomonads, and other Gammaproteobacteria, was found for the *E. coqui* population at Charco Azul. Members of Pseudomonadaceae, among others, were found abundant in the skin of another direct-developing frog in Panama, at a site were the pathogenic chytrid fungus (*Bd*) was present. Whereas a more diverse array of differentially abundant taxa was found for the *Bd* free sites (such as members of Alphaproteobacteria, Saprospirae and Sphingobacteriia), as we found here for the Quebrada site (Rebollar et al., 2016). Although we did not test for *Bd* presence in this study, this pathogen is enzootic to *Eleutherodactylus* populations at elevations >600 masl in Puerto Rico (as in Charco Azul), and has been detected in the Sierra de Cayey where this study was conducted (Burrowes et al., 2008). Pseudomonads might be related to *Bd* presence, but environmental conditions that enhance *Bd* occurrence could also favor particular bacterial taxa. In the future, advancing studies on the function of metabolites produced by these OTUs will shed light toward their potential protective role against pathogens affecting the skin of hosts (Daskin et al., 2014; Woodhams et al., 2014).

### Phylogeny Effect

Host phylogeny was not a strong factor shaping skin microbiota in these closely related hosts. The small number of host species studied could have hindered our chances to detect congruence with phylogeny, especially as locality and host species effects on skin microbiota were stronger. This is supported by the fact that removing the most ecologically distinct species, *E. cooki*, improved correlation between host phylogenetic distances and unweighted Unifrac distances (but not with weighted distances). A smaller influence of host phylogeny on skin microbiota, compared to other factors, was also found in studies at others amphibian taxonomic levels (Bletz et al., 2017a; Hernández-Gómez et al., 2017). In these studies however, the effect of variables like locality or host ecology could also be confounded (see also Bird et al., 2018). Varying degrees of congruence between microbiota and host phylogeny have been found in mammals and insects (Ley et al., 2008; Ochman et al., 2010; Yildirim et al., 2014; Abdul Rahman et al., 2015; Brooks et al., 2016). Skin is more constantly in contact with the environment than other body parts (e.g., gastrointestinal tract), and this might facilitate colonization of new microbes, hindering any phylogenetic congruence, while emphasizing locality effects. Another explanation for the low phylogenetic signal found, may be that we analyzed the whole bacterial community of *Eleutherodactylus* skin. A recent study found that host phylogeny influenced only certain bacterial taxa on mammals, having a stronger effect on more recent gut bacterial lineages while ancient lineages were influenced mostly by host diet (Groussin et al., 2017). Comparing patterns at different geographic and phylogenetic scales (for both host and bacteria), and among amphibian groups of different ecological habits, will help discern circumstances in which the effect of host phylogeny may be important.

## Conclusion

Our findings support previous evidence that skin bacterial communities in amphibians differ from environmental communities, and that both environment and host associated factors influence these communities, albeit in different ways. For this group of terrestrial, tropical frogs, locality had the strongest effect, mostly in skin bacteria composition while host species, although subtler, influenced mainly bacterial community structure. In addition, the extent of ecological specialization can affect host skin microbiota as observed for the caves/rock dweller *E. cooki* and the generalist *E. coqui*, respectively. Thus, the potential for skin microbiome to be similar among ecomorphs in repetitive radiations, like the *Eleutherodactylus* in the Caribbean (Dugo-Cota et al., 2019), will depend in the degree of specialization, and most likely will be observable in the structure versus the composition of bacteria. Our study shows that factors other than phylogeny and microhabitat use may influence specific host’s microbiome in a small clade of *Eleutherodactylus*. This was also found for co-occurring congeneric frogs in Australia (Christian et al., 2018), highlighting that factors influencing host-specificity of skin bacterial communities are still poorly understood. Further studies on the physiological and immunological aspects of these frogs will help disentangle the intrinsic factors involved in the maintenance of species-specific skin-associated microbial communities.

Considering that amphibians are presently being devastated by a pathogenic chytrid fungus (Skerratt et al., 2007; Scheele et al., 2019), and that their skin bacteria have shown a potential role in defense against this pathogen (Harris et al., 2006; Becker et al., 2015a, b; Knutie et al., 2017), it is vital to study the influence of underlying factors determining the structure and composition of bacterial communities in host’s skin. From a conservation perspective, advancing this line of research will help guide effective managements strategies for species persisting under enzootic pathogen conditions, and determine when environmental augmentation of specific bacterial OTU’s could mitigate the effect of pathogens in wild amphibian populations.

## DATA AVAILABILITY STATEMENT

The datasets generated for this study can be found in the SRA repository under Bioproject PRJNA553070 and Submission ID SUB5920343.

## ETHICS STATEMENT

This research was performed under the Department of Natural Resources and Environment of Puerto Rico permission (DRNA 2016-IC-030) and the Institutional Animal Care and Use Committee at University of Puerto Rico approval (Protocol 01002-05-272014).

## Author Contributions

LG-R and PB collected the field data. LG-R performed the sequence processing and data analysis. MD-B and PB contributed with equipment and reagents. All authors designed the project and contributed to writing and revision of the manuscript.

## Conflict of Interest

The authors declare that the research was conducted in the absence of any commercial or financial relationships that could be construed as a potential conflict of interest.
