# Supplementary figures and images for: The Skin Microbiota of Eleutherodactylus Frogs: Effects of Host Ecology, Phylogeny, and Local Environment
Source: Front Microbiol. 2019 Nov 6;10:2571. doi: 10.3389/fmicb.2019.02571 (PMC6856660; doi:10.3389/fmicb.2019.02571)

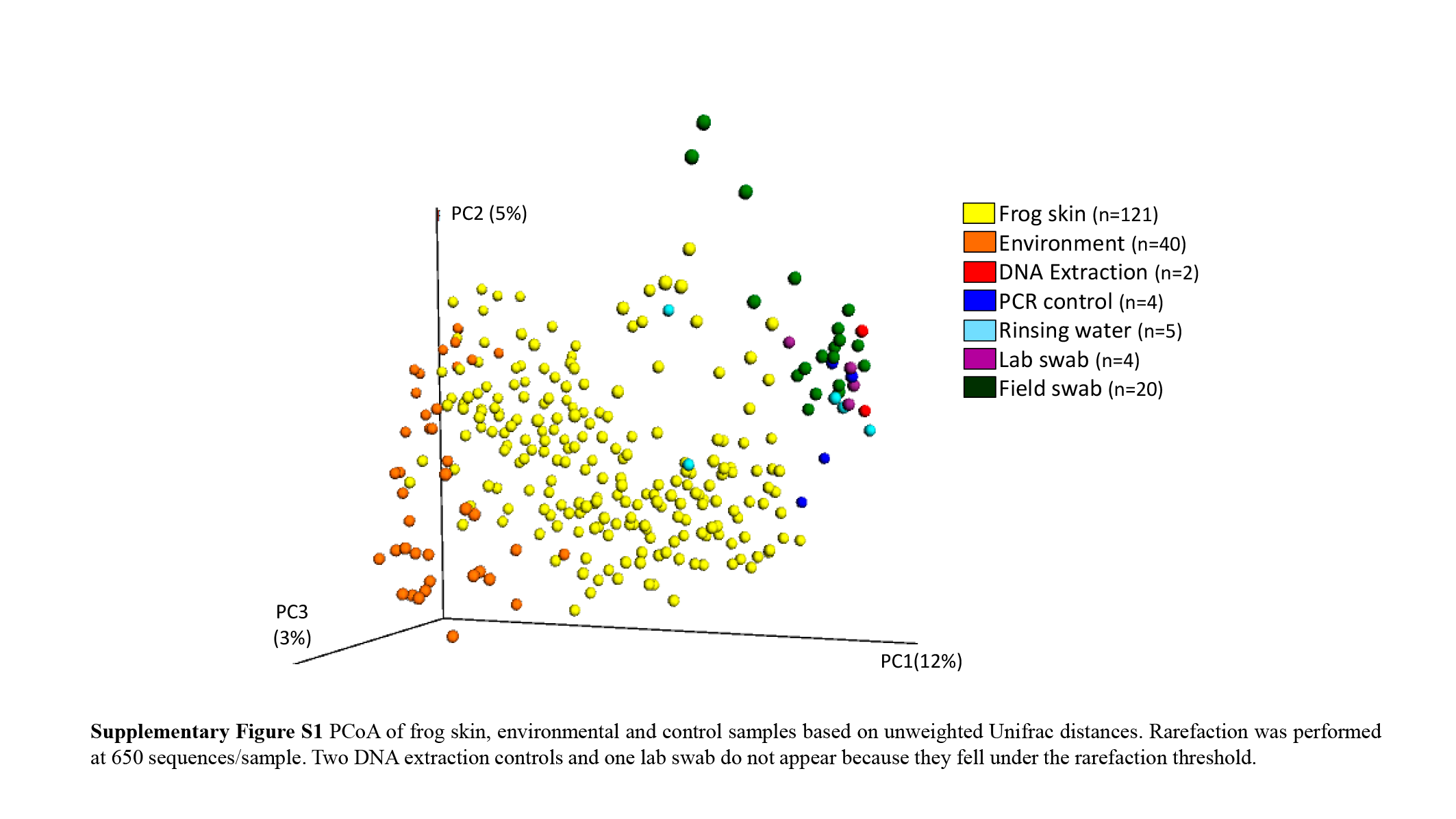

Supplement: Supplementary file 10 [file Image_1.PNG]

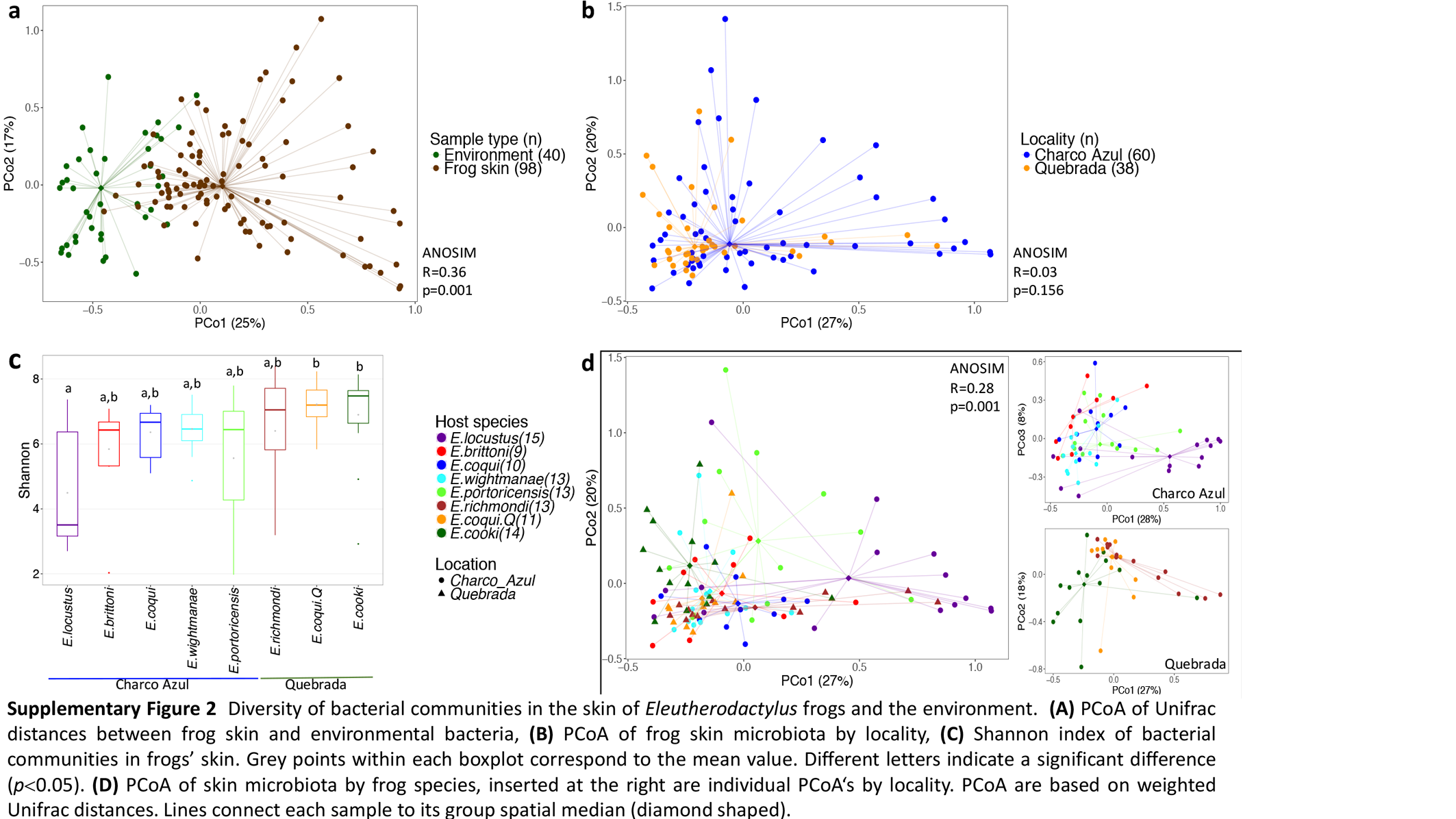

Supplement: Supplementary file 11 [file Image_2.PNG]

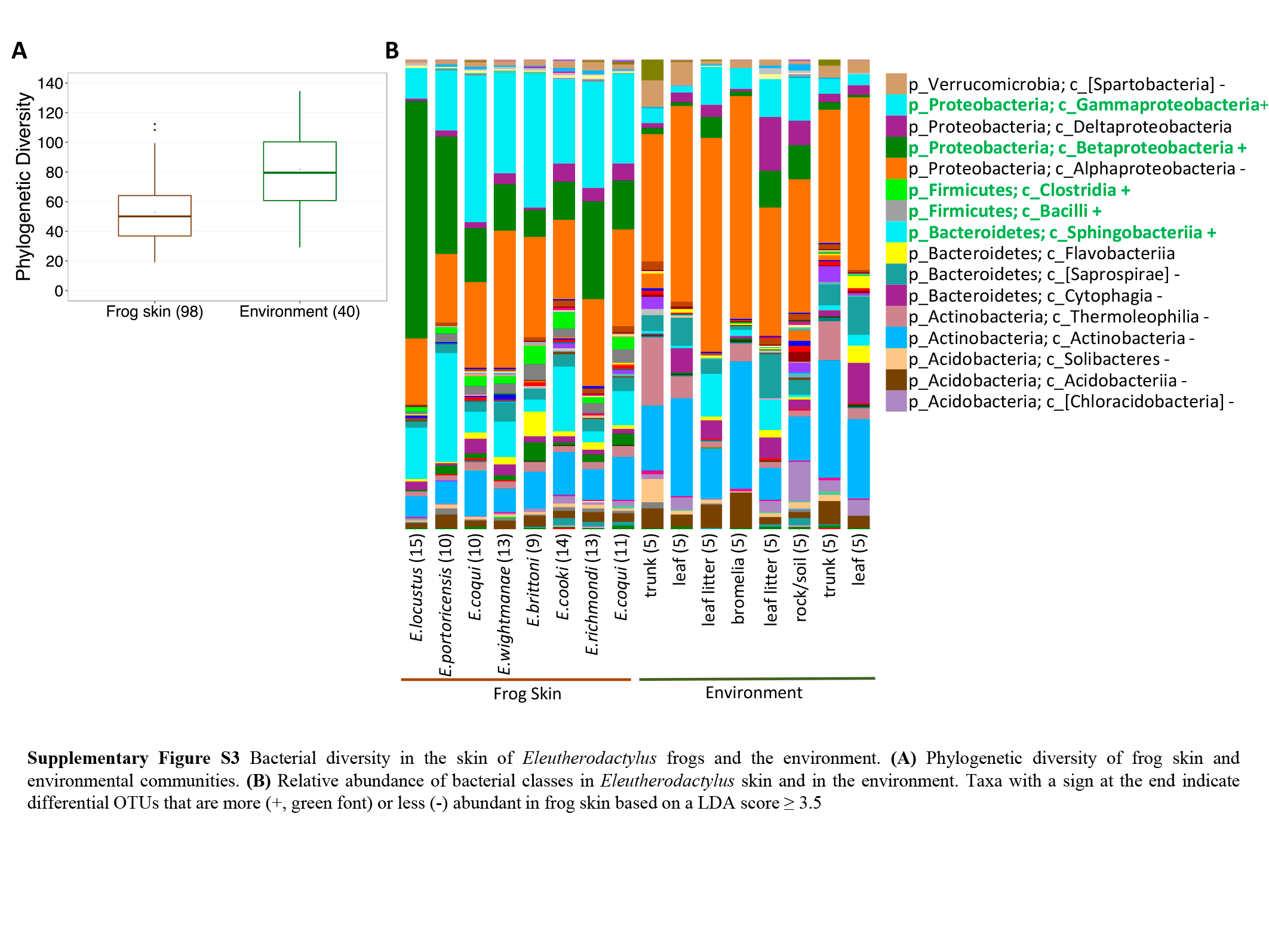

Supplement: Supplementary file 12 [file Image_3.PNG]

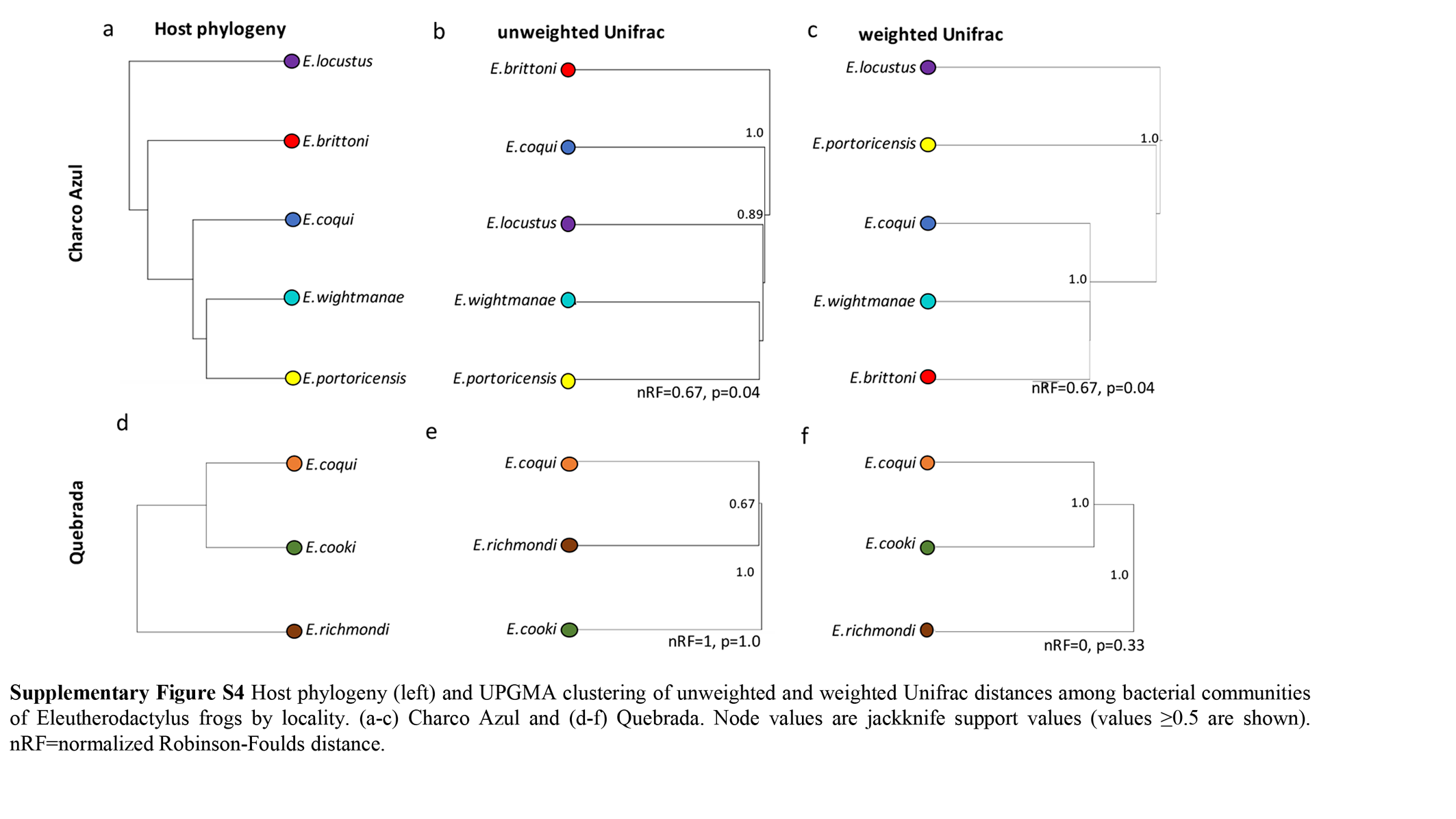

Supplement: Supplementary file 13 [file Image_4.PNG]
